# *fam20*C participates in the shell formation in the pearl oyster, *Pinctada fucata*

**DOI:** 10.1038/s41598-018-21797-w

**Published:** 2018-02-23

**Authors:** Jinzhe Du, Chuang Liu, Guangrui Xu, Jun Xie, Liping Xie, Rongqing Zhang

**Affiliations:** 1 Institute of Marine Biotechnology, School of Life Sciences, Tsinghua University, Beijing, 100084 China; 2 Department of Biotechnology and Biomedicine, Yangtze Delta Region Institute of Tsinghua University, Jiaxing, Zhejiang Province 314006 China; 3 Protein Science Laboratory of the Ministry of Education, Tsinghua University, Beijing, 10084 China

## Abstract

Kinase-family with sequence similarity 20, member C (Fam20C) is a protein kinase, which can phosphorylate biomineralization related proteins in vertebrate animals. However, the function of Fam20C in invertebrate animals especially the role in biomineralization is still unknown. Herein, we cloned the cDNA of *fam20*C from the pearl oyster, *Pinctada fucata*. It is showed that the expression of *fam20*C in the mantle edge was much higher than other tissues. *In situ* hybridization showed that *fam20*C was expressed mostly in the outer epithelial cells of the middle fold, indicating it may play important roles in the shell formation. Besides, *fam20*C expression increased greatly in the D-shape stage of pearl oyster development, when the shell was first formed. During the shell repair process, the expression level of *fam20*C increased 1.5 times at 6 h after shell notching. Knockdown of *fam20*C *in vivo* by RNA interference resulted in abnormally stacking of calcium carbonate crystals at the edges of nacre tablets, showing direct evidence that *fam20*C participates in the shell formation. This study provides an insight into the role of kinase protein in the shell formation in mollusk and broaden our understanding of biomineralization mechanism.

## Introduction

Biomineralization is a process that minerals are formed by organisms^1^. Pearl oyster, *Pinctada fucata* is one of the most important cultured pearl species in China, and is also a good model species to study biomineralization^2^. The shell of *P*. *fucata* includes two layers, an inner nacreous aragonite layer, and an outer prismatic calcite layer. Both layers are composed of more than 95% calcium carbonate and <5% of organic macromolecules especially proteins, which are important for crystal nucleation, polymorphism, orientation and organization during shell formation^3,4^. Previous studies have reported many matrix proteins from shells. For instance, acidic matrix proteins with cation binding properties are known as important proteins in calcium carbonate crystallization and shell formation^5–7^. Several matrix protein domains have been identified including carbonic anhydrase domain in nacrein^8^ and N66^9^, lectin domain in perlucin^10^ and pontin protein domain in dermatopontin^11^.

It is noted that some matrix proteins have post-translational modification such as glycosylation, phosphorylation and sulfation, which are crucial for their functions^12^. Phosphorylation is one of the most widespread post-translational modifications of proteins and also occurs in the organic matrix of biominerals^13,14^. Kinase is a series of evolutionary conserved enzyme, playing important roles in regulating cellular events by phosphorylating substrates^15^. Fam20C, also called dentin matrix protein 4, is a kind of kinase encoded by *fam20*C gene in *homo sapiens*^16–18^. Fam20C family was first identified in the process of hematopoietic stem cell differentiation into bone marrow cell^16^. In recent years, Fam20C is found to be a serine protein kinase located in Golgi and is able to phosphorylate casein and SIBLING (Small integrin-binding ligand, the N-linked glycoproteins) family by phosphorylating S-x-E motif^19^. The finding of Fam20C locating in Golgi proves that the ATP-dependent protein phosphorylation can occur in secreted pathways^20,21^. It is also revealed that Fam20C can modulate the differentiation of mesenchymal stem cells into odontoblast and regulate enamel mineralization^17^. In addition, Fam20C was highly expressed in dental tissues in mice and was highly expressed in odontoblast during the developing process of mice teeth, indicating that the Fam20C was closely related to mineral formation^17^. These studies confirmed that Fam20C was closely involved in the biomineralization process of vertebrate animals. However, the function of Fam20C in mollusk and the role of kinase in mollusk shell formation is poorly understood.

In pacific oyster *C. gigas*, tissue-specific microarray analysis showed that *fam20*C was highly expressed in the mantle^22^. The *Lottia gigantea* shell matrix phosphoproteome revealed that one third of phosphorylation sites were at the serine site of S-x-E motif, compared with 24% in human secreted phosphoproteins^23^. Recently, a dentin-matrix protein-like (DMP-like), exhibiting a remarkable Fam20C domain was detected in two freshwater mussels unionoid proteomes^24^. cfMSP-1, an extremely acidic matrix protein involved in shell formation of the scallop *Chlamys farreri*, had eight S-D-E motifs, which were possibly phosphorylated by Fam20C^25^. Despite these studies, no direct evidence has been given on the role of Fam20C in shell formation. Therefore, in this study, we cloned the cDNA of *fam20*C from the pearl oyster *P*. *fucata* and studied the tissue-specific distribution as well as the expression profiles during different development stages. In addition, shell notching experiment and RNA interference were performed to investigate the role of Fam20C in biomineralization *in vivo*.

## Methods

### Ethics statement

This study was approved by the Animal Ethics Committee of Tsinghua University, Beijing, China.

### Experimental animals

All pearl oysters *P*. *fucata* used in this study were collected from Zhanjiang, Guangdong province of China and were cultured at 20 degrees centigrade in artificial seawater (3% salinity).

### Tissue collection and preparation

Different tissues were extracted from the control or treated oysters. Then the tissues were immediately flash-frozen and were powdered in liquid nitrogen for further experiments. Especially, the samples of different developmental stages including oosperm stage, trochophore stage, D-shape stage, umbonal stage and juvenile stage were stored in RNAlater RNA stabilization reagent (Qiagen) and were collected from Zhanjiang, Guangdong province of China.

### Total RNA extraction

Total RNA was extracted using Trizol reagent (Life technologies) following the manufacturer’s instruction. RNA integrity and purity were checked by 1.2% agarose gel electrophoresis and an UV/visible spectrophotometer (Ultrospec 3000, Amersham). RNA concentration was determined by NanoDrop 2000 (Thermo Scientific).

### cDNA library construction

cDNA library was prepared by reverse transcription-PCR of the total RNA with GoScript^TM^ Reverse Transcription System (Promega) following the manufacturer’s instructions.

### Full-length cDNA cloning by RACE

A conserved DNA sequence of *fam20*C gene was acquired by alignment between transcriptome of *P*. *fucata* and human *fam20*C gene sequence by Tagliabracci *et al*.^19^. The full-length cDNA of *fam20*C was cloned from the mantle total RNA using SMART^TM^ RACE cDNA Amplification kit (Clontech) following the instructions. RACE PCR was performed using primers named as Fam20C-3′RACE and Fam20C-5′RACE. Confirmation PCR ‘rimers were named as Fam20C-confirm-F and Fam20C-confirm-R. All primers used in this study were listed in Table S1.

### Nucleotide and amino acid sequences analysis

Blast of sequences was attempted using Blastp and Blastn searches against NCBI database (http://blast.ncbi.nlm.nih.gov/Blast.cgi). The theoretical molecular mass, isoelectric point and amino acid composition of the proteins were computed using ProtParam from the EXPASY online server. Conserved domains were predicted using SMART (http://smart.embl-heidelberg.de/) and InterproScan (http://www.ebi.ac.uk/interpro/search/sequence-search). The alignment of sequences from different species was performed by online software Clustal Ω (http://www.ebi.ac.uk/Tools/msa/clustalo/). Signal peptide was predicted by online tool signal P4.1 (http://www.cbs.dtu.dk/services/SignalP/).

### Gene expression analysis by real-time PCR

Real-time PCR was performed using StepOnePlus^TM^ Real-time PCR systems (Applied Biosystems) with SYBR^®^ Premix Ex Taq^TM^ II Kit (Takara), with GAPDH as an internal reference due to its relatively stable expression in different tissues. Templates were cDNA libraries of different tissues. Primers were designed according to *fam20*C sequence named as qFam20C-F and qFam20C-R. The control primers were named as qGAPDH-F and qGAPDH-R. Different gene expression level was calculated using 2^−ΔΔCt^ method by Livak and Schmittgen^26^.

### *In situ* hybridization experiment

The mantle of pearl oyster was removed and was immediately fixed overnight in 4% paraformaldehyde containing 0.1% diethyl pyrocarbonate (Sigma) and was then washed in 0.1 M PBS. Washed sample was soaked in 20% sucrose solution at 4 degrees centigrade. Then frozen mantle section was prepared for *in situ* hybridization. The DNA fragments were amplified with the primer pair Fam20C-F and Fam20C-R and were inserted in multiple cloning sites of vector pEASY-T3 (Promega). Synthesized RNA probe was produced using DIG RNA Labeling Kit (Roche) with T7 and SP6 RNA polymerase for the sense and anti-sense probe respectively. *In situ* hybridization was carried out using Enhanced Sensitive ISH Detection Kit II (BOSTER). To avoid false positive signals, the hybridization temperature was increased to 58 degrees centigrade.

### Shell notching experiment

The shell notching of pearl oysters was performed as described by Mount *et al*.^27^. Pearl oysters were randomly divided into eight groups with five animals each and were cultured in seawater tanks. At 0, 6, 12, 24, 36, 48, 72, and 96 h after shell notching, oysters were killed and mantle tissues were collected. The methods of RNA extraction, reverse transcription and real-time PCR were mentioned above.

### Gene silencing *in vivo* by RNA interference (RNAi)

RNAi experiment was conducted according to the method by Suzuki *et al*.^28^ with some modifications. Firstly, DNA segments were produced by PCR. The primers were dsFam20C-F and dsFam20C-R; dsGFP-F and dsGFP-R. *fam20*C gene and *gfp* segment were produced from the mantle cDNA and pEGFP-C1(Clontech) respectively. Next, dsRNA was transcribed from DNA segment by RiboMaX^TM^ Large Scale RNA Production System (T7) Kit (Promega) following manufacturer’s instructions. Then the synthesized dsRNA products were diluted to 80 μg/100 μL and 160 μg/100 μL by 0.1 M PBS. 100 μL of dsRNA was injected into four oysters for RNAi and 0.1 M PBS was injected as the control. All oysters were killed after six days and mantle tissues were collected. The shells were sampled after 6 days after RNAi experiment because it usually took about 6 days for the growth of new shells. We want to check the effect of *fam20*C knockdown on the new generated shells. Therefore, the time point of 6 days were chosen and the method has been used for RNAi experiment of matrix proteins^28^. The methods of RNA extraction, reverse transcription and real-time PCR were mentioned above. The shells were collected for further scanning electron microscope (SEM) observation.

### SEM observation of shells

Collected shells were rinsed in MiliQ water 3 times before air drying in an incubator. Cleaned shell was sprayed gold nanoparticles for 60 s before observation. The morphologies of the inner surface of shells were examined by SEM (FEI Quanta 200, 15 keV).

### Data accessibility

Nucleotide sequence of *fam20*C of *P*. *fucata* is available in the GenBank database under the accession numbers MF785096.

### Statistical analysis

Multigroup comparisons of the means were carried out by one-way analysis of variance (ANOVA) test with post hoc contrasts by Bonferroni test (IBM SPSS Statistics 22 software). The statistical significance for all tests was set at P < 0.05. (*P < 0.05, **P < 0.01).

## Results

### Cloning and bioinformatics analysis of *fam20*C

**Figure 1:**
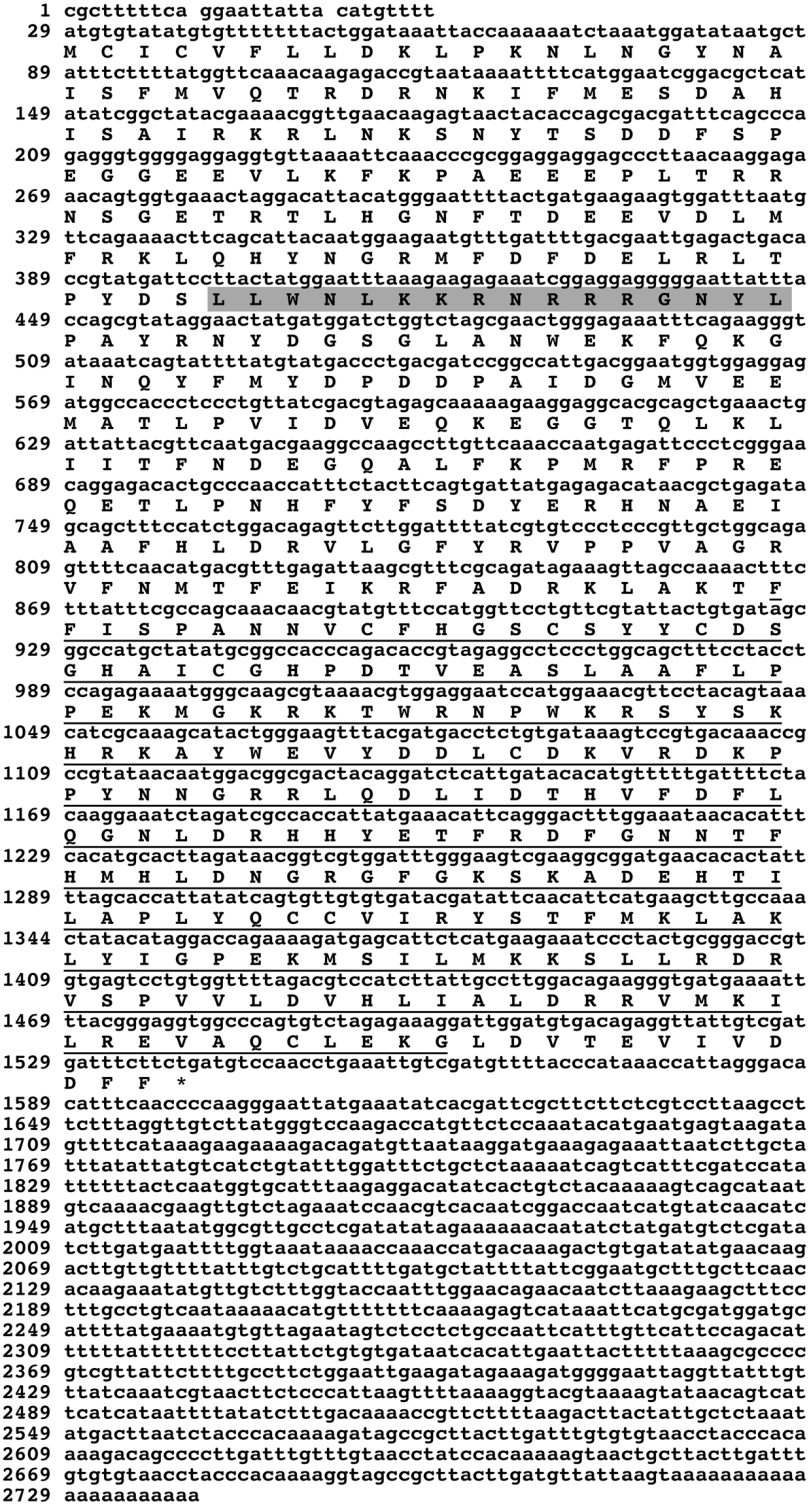
Full-length sequence of cDNA and deduced amino acid sequence of *fam20*C of the pearl oyster, *P*. *fucata*. The shaded region is low complexity region (LCD) and the underlined region is kinase domain PFAM.

**Figure 2:**
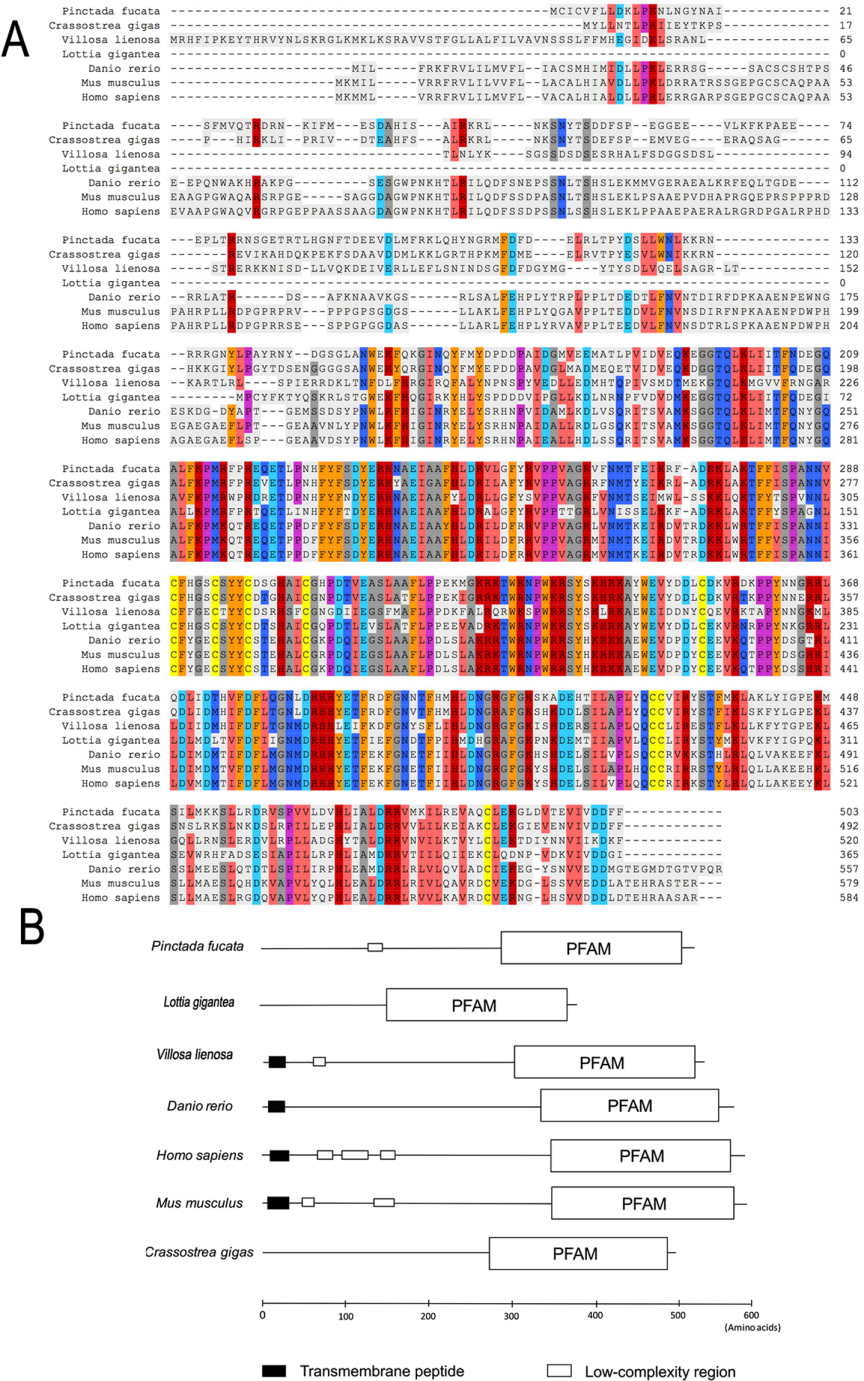
Bioinformatics analysis of *fam20*C gene. (**A**) Homology comparison of Fam20C protein from different species. (**B**) The predicted functional domains of Fam20C among different species.

Based on the homology of *fam20*C gene in different species, we found a highly conserved DNA sequence in the mantle transcriptome of *P*. *fucata*. Then, we cloned *fam20*C full-length sequence from the mantle cDNA library and obtained a 2739-bp transcript including a 5′-untranslated region of 28 bp, an open reading frame of 1512 bp encoding a protein containing 503 amino acids, and a 3′-untranslated region of 1199 bp (Fig. 1). The deduced mature protein had a molecular mass of 58.9 kDa and the theoretical isoelectric point was 8.36. After retrieving sequences of several important species including *Danio rerio*, *Mus musculus*, *Homo sapiens*, *Lottia gigantean*, *Villosa lienosa* and *Crassostrea gigas* from NCBI data base, a sequence alignment analysis was performed by Clustal Ω. It is showed that *fam20*C had more similarity at C-ends among different species, but sequences at N-ends were species-specific (Fig. 2A). The human and mouse *fam20*C gene were more complex than other species. *fam20*C of *P*. *fucata*, *Lottia gigantean* and *Crassostrea gigas* lacked transmembrane peptide, while all three vertebrates species and *Villosa lienosa* had transmembrane peptides. Like human and mouse, *fam20*C of *P*. *fucata* had a low complexity region from amino acid 125 to 140 (Fig. 1), which may be involved in flexible binding^29^. Fam20C among different species had a conserved function domain with their own features (Figs 1, 2B). SignalP 4.1 showed that Fam20C of *P*. *fucata* had no signal peptide (Fig. S1), which conformed to the result of domain prediction, indicating that Fam20C of mollusks may have a different function compared with well-studied Fam20C of vertebrates.

### The distribution of *fam20*C expression in different tissues

**Figure 3:**
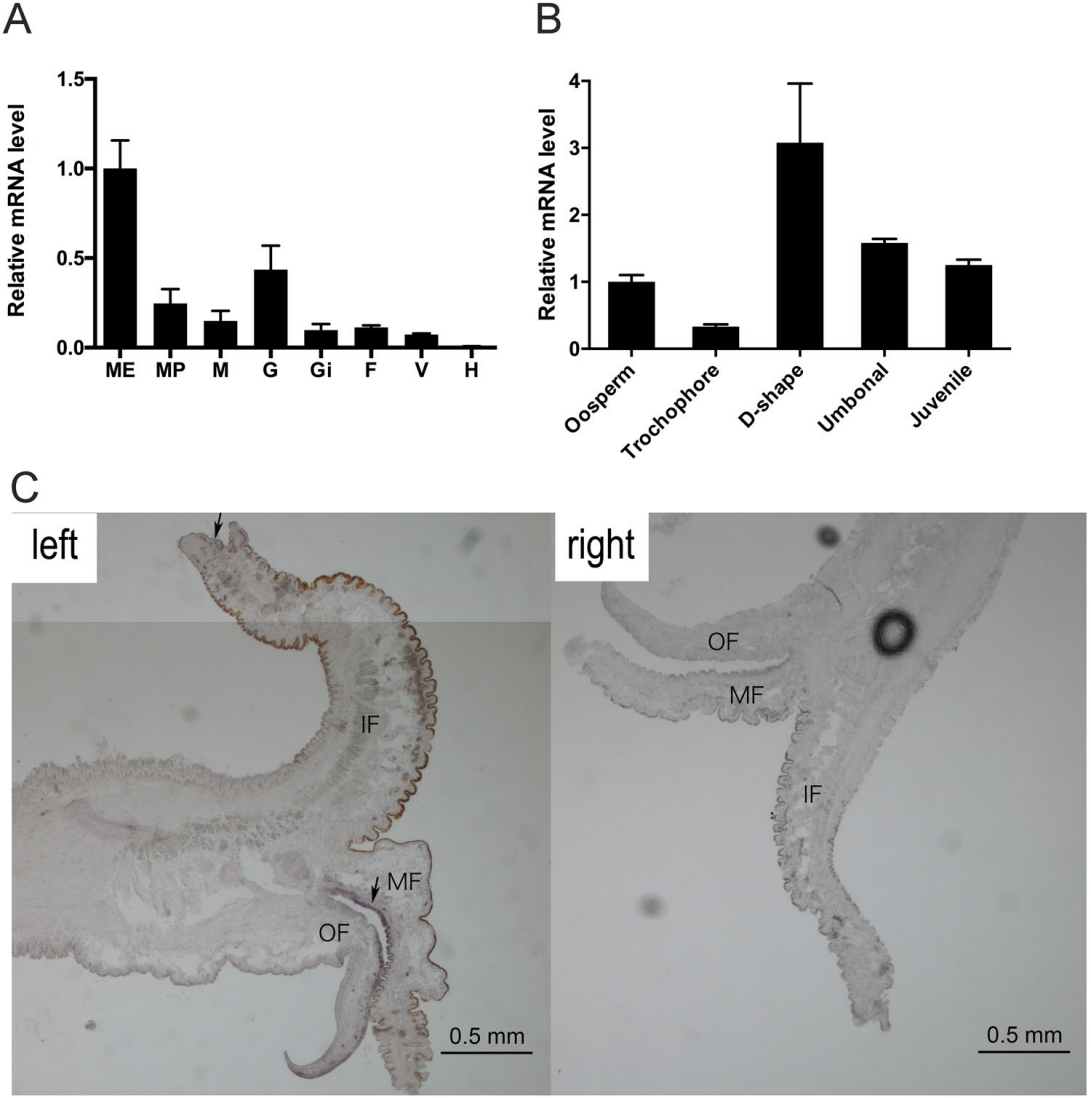
Expression and distribution of *fam20*C mRNA. (**A**) Relative gene expression levels of *fam20*C in different tissues (From left to right: mantle edge, mantle pallial, adductor muscle, gonad, gill, foot, visceral mass and hemocytes). (**B**) *fam20*C expression levels varies during five development stages. (**C**) *In situ* hybridization of *fam20*C mRNA in the mantle of pearl oyster. Among the three folds of the mantle, hybridization signals (arrows) were observed at the outer epithelial cells of the middle fold and the inner epithelial cells of the inner fold of the mantle (left). No hybridization signal is apparent in the control section stained with the antisense probe (right). OF, outer fold; MF, middle fold; IF, inner fold. The error bars are the standard deviation of four independent samples.

To determine the distribution of *fam20*C mRNA in different tissues, we conducted real-time PCR. *fam20*C mRNA was expressed in all tissues except hemocytes (Fig. 3A). However, the expression level was much higher in mantle edge than in gonad, mantle pallial and adductor muscle. Moreover, *in situ* hybridization was carried out to study the spatial distribution of *fam20*C in the mantle. Strong hybridization signals were detected at the outer epithelial cells of the middle fold, while a faint signal were detected at the inner epithelial cells of the inner fold of the mantle (Fig. 3C left). Meantime, no obvious signal was found using the control probe for hybridization (Fig. 3C right).

### *fam20*C expression level varies during development stages

In order to investigate the function of *fam20*C gene in the larval development, we conducted real-time PCR to study the gene expression during the development process. *fam20*C expression increased by 3 times in D-shape stage than in oosperm and the expression level dropped in umbonal stage and juvenile stage (Fig. 3B). The D-shape stage is the time when the shell is first formed, suggesting that *fam20*C is closely related to shell formation.

### The role of *fam20*C in biomineralization *in vivo*: shell notching and RNA interference experiment

**Figure 4:**
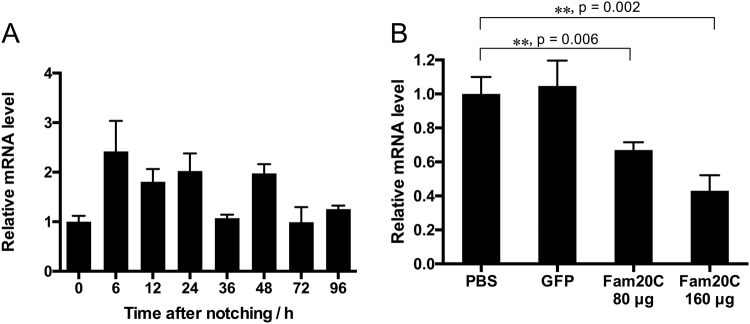
Shell notching and RNAi of *fam20*C. (**A**) Response of the *fam20*C gene during shell repairing after shell notching. 0 h after shell notching groups had a relative value of 1.0. (**B**) Relative *fam20*C gene expression level after RNAi. PBS-injected groups had a relative value of 1.0. The non-target control was *gfp* dsRNA injected groups. Two experimental groups were injected with *fam20*C dsRNA with the dose of 80 μg and 160 μg. The error bars are the standard deviation of four independent samples. The stars represent a significant (**p < 0.01) difference compared with the PBS-injected groups (p = 0.006 and p = 0.002 compared to the PBS-injected group for 80 μg and 160 μg group, respectively). In contrast, there is no significant difference between PBS-injected group and gfp-injected group. The method used is One-way ANOVA performed by SPSS Statistics 22 software.

To investigate the *fam20*C functions in shell formation, we conducted shell notching assays to induce shell repairing. The expression of *fam20*C increased 1.5 times 6 h after shell notching and maintained a high level until 36 h (Fig. 4A). At 36 h after shell notching, *fam20*C expression decreased to the initial level. Regenerated shell was firstly found 3–6 days after shell notching (Fig. S2). During the shell repair process, the gene expression was not greatly changed, but it had a tendency of increasing and then decreasing to the normal state. These results may be related to the phosphorylation of proteins during the shell repair process, which needed further study.

**Figure 5:**
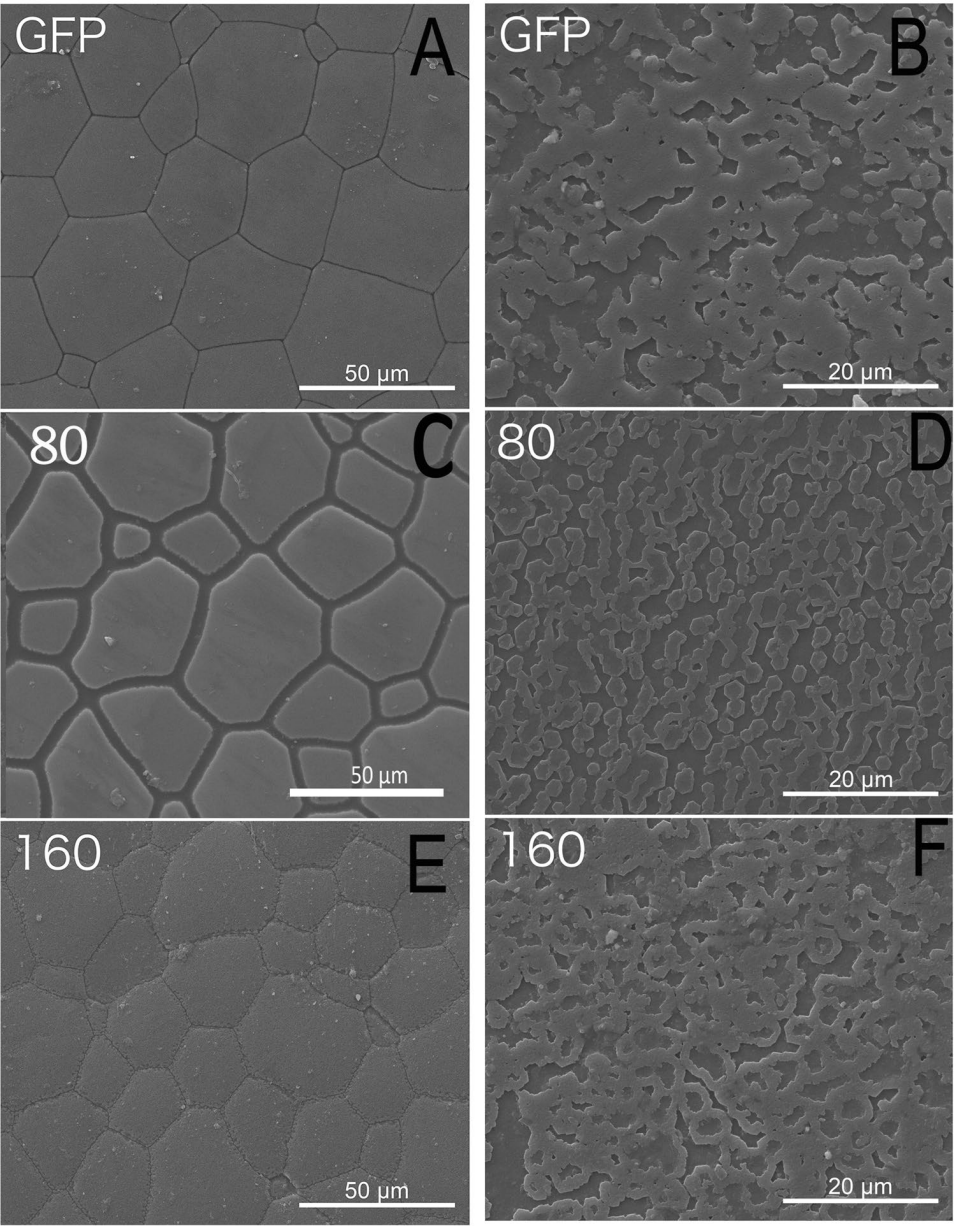
SEM observation of shell surfaces after RNAi. (**A**) The prismatic layer and the nacreous layer (**B**) of *gfp* dsRNA-injected group. (**C**) The prismatic layer and the nacreous layer (**D**) of 80 μg *fam20*C dsRNA-injected group. (**E**) The prismatic layer and the nacreous layer (**F**) of 160 μg *fam20*C dsRNA-injected group.

The function of *fam20*C during the shell formation was further analyzed by RNAi. *fam20*C dsRNA were injected into adductor muscles. For the control, *gfp* dsRNA and PBS were injected. *fam20*C expression in the PBS-injected and *gfp* dsRNA-injected groups were similar. Compared to the PBS-injected group, *fam20*C expression level decreased by approximately 30% in the group injected with 80 μg *fam20C* dsRNA and 50% in the group injected with 160 μg *fam20*C dsRNA (Fig. 4B). The surfaces of the shells were observed by SEM. In the *gfp* dsRNA-injected group, the prismatic layer showed normal prism structures (Fig. 5A) and the nacreous layer showed a normal pattern like “brick wall” accumulated by small nacreous tablets (Fig. 5B). After injecting 80 μg *fam20*C dsRNA, cavities started to appear in the center of the nacre tablets and they were covered with randomly accumulated crystals (Fig. 5D). When the concentration increased to 160 μg, randomly accumulated crystals became more interconnected and thick (Fig. 5F). The prismatic layer was not severely disturbed as the nacreous layer, while the edges of prisms became slightly unclear when injected 80 (Fig. 5C) or 160 μg *fam20*C dsRNA (Fig. 5E). Moreover, more images of shell phenotypes from the control animals and treatment animals (both 80 and 160 treatments) have been provided in the Fig. S3 (SEM images of prismatic layers) and Fig. S4 (SEM images of nacreous layers). In our experiment, we mixed the samples so three images for each group were shown and we believed that they should represent the typical results of each experiment based on our groups’ experience in similar experiment^30^. As we can see in Fig. S3, no significant difference of prismatic layers have been observed in the *gfp*-injected group and *fam20*C dsRNA-injected group. In contrast, significant difference of nacreous layers have been observed in the *gfp*-injected group and *fam20*C dsRNA-injected group (Fig. S4). Therefore, to statistically analyze the effects after RNAi injection, we have quantified the diameter of overgrowth crystals on nacre (red arrows in Fig. S4B and C1). The statistics showed that the average diameters of overgrowth crystals on nacre tablets of 80 μg *fam20*C dsRNA-injected group and 160 μg *fam20*C dsRNA-injected group were 0.44 μm and 1.72 μm, respectively, both significantly larger than that of *gfp*-injected group (Fig. S4D). Moreover, we estimated the normal hexagon nacre tablets in different groups and found that the percent of normal hexagon nacre tablets were decreasing with the increase of *fam20*C dsRNA injection (Fig. S4E).

## Discussion

Recent studies were mainly focused on the functions of matrix proteins in shell formation. Although the roles of matrix proteins in crystal growth have been gradually revealed, more upstream regulating proteins should be studied. Fam20C could be one of the candidates due to its kinase property. We showed that Fam20C was a highly conserved protein among different species. Interestingly, Fam20C lacked signal peptides and transmembrane peptides in most invertebrates, which has implications for understanding the different function of Fam20C.

RT-PCR results indicated that Fam20C may have diverse functions besides mineralization function. The distribution in gonads suggested that Fam20C may be related to development and cell differentiation. Its abundant distribution in mantle edge indicated that it plays important roles in shell formation. As it is known, the mantle is the most important tissue involved in biomineralization and different regions of mantle are responsible for periostracum, prismatic layer, nacreous layer according to their expression and secretion of matrix proteins or regulating proteins. Previous studies revealed that the proteins such as KRMP family^31^ expressed in mantle edge were responsible for prismatic layer formation, and proteins expressed in mantle pallial such as MSI60^32^ and nacrein^8^ constructed the nacreous layer. The proteins from the outer epithelial cells of the middle fold, such as tyrosinase^31^ were responsible for periostracum formation. The proteins from the inner epithelial cells of the inner fold, such as MSI7^33^, participated in the formation of nacreous layer. Based on that, Fam20C may be related to the formation of all shell layers. However, recent studies showed that protein secreted from specific regions cannot determine its function in biomineralization from the studies of PfN23^34^ and PfN44^35^. The relation between protein locating regions and shell layer formation needs to be studied further. Previous studies revealed that Prodissoconch I, composed of amorphous calcium carbonate, forms at the early D-shaped stage; whereas Prodissoconch II, which contains aragonite and calcite, appears in the late D-shaped and umbonal stages^36^. Dissoconch shell, the original shell with an inner nacreous layer and an outer prismatic layer, forms at the juvenile stage and grows throughout life^30^. The distribution of gene expression in different development stages showed that Fam20C had a close connection with Prodissoconchi I formation and may be involved in larval development due to its high expression in gonad.

Shell notching experiments were carried out to detect the *in vivo* effect of *fam20*C during shell repair process. *fam20*C expression responded positively during the shell regeneration process after shell notching and the expression level of *fam20*C then decreased gradually to a relatively stable value. The RNAi experiment is an effective way to detect functions of matrix proteins *in vivo*, such as Pif, PfN23, and PfN44^28,34,35^. Knockdown of *fam20*C affected the shell formation especially the nacre formation.

In conclusion, we provided evidences that *fam20*C participated in shell formation. Regulation of shell biomineralization is a sophisticated biological process, which requires participation of many cells and proteins. Shell formation is not regulated by one protein. We assume that Fam20C is a regulator in biomineralization and it acts as a controller rather than an inhibitor or enhancer. However, whether Fam20C phosphorylates matrix proteins in the shell formation process still remain unexplored and warrants further study. Nevertheless, this study provides a novel understanding of the function of *fam20*C in mollusk, which has implications for understanding the regulation mechanisms of shell formation as well as for the development of nacre-like materials. Moreover, it lays foundation for future study about the relationship between regulating proteins and matrix proteins, which can give us more information about biomineralization from a new perspective.

## Electronic supplementary material

Supplementary information
