# Supplementary material for: fam20C participates in the shell formation in the pearl oyster, Pinctada fucata
Source: Sci Rep. 2018 Feb 23;8:3563. doi: 10.1038/s41598-018-21797-w (PMC5824888; doi:10.1038/s41598-018-21797-w)
Supplement: Supplementary file 1 — Supplementary information [file 41598_2018_21797_MOESM1_ESM.doc]

*fam20*C participates in the shell formation in the pearl oyster, *Pinctada fucata*

Jinzhe Du^1,2#^, Chuang Liu^2#^, Guangrui Xu^2^, Jun Xie^1^, Liping Xie^1^, and Rongqing Zhang^1, 2*^

^1^ Institute of Marine Biotechnology, School of Life Sciences, Tsinghua University, Beijing 100084 China

^2^ Department of Biotechnology and Biomedicine, Yangtze Delta Region Institute of Tsinghua University, Jiaxing, Zhejiang Province, 314006, China

^#^Jinzhe Du and Chuang Liu contributed equally to this work.


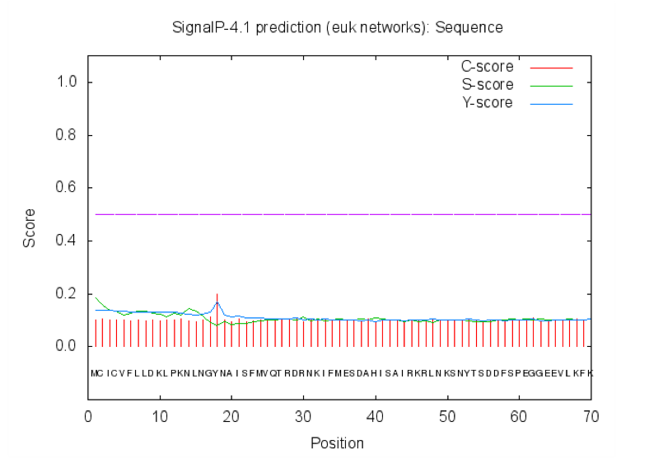


**Figure S1.** Signal peptide analysis of Fam20C by SignalP 4.1


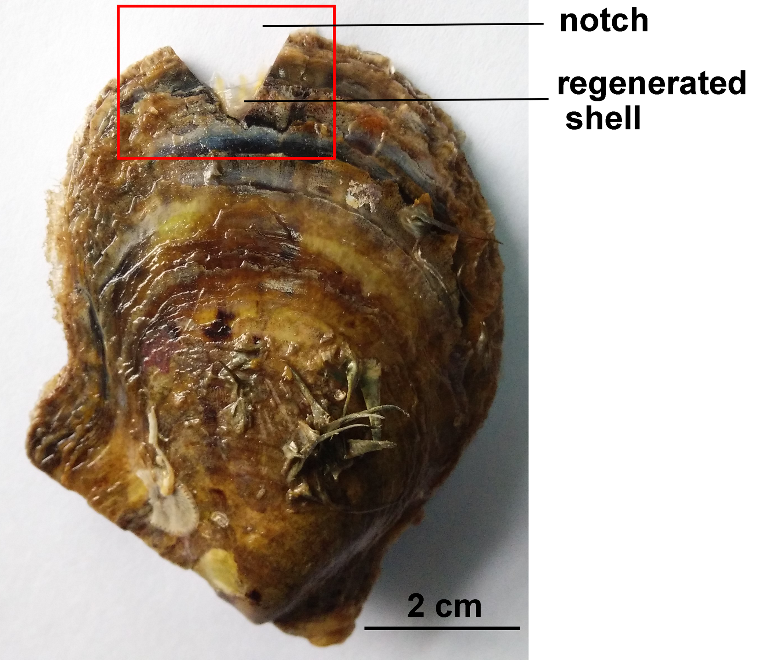


**Figure S2.** Regenerated shell of *Pinctada fucata* after shell notching for about 3 days


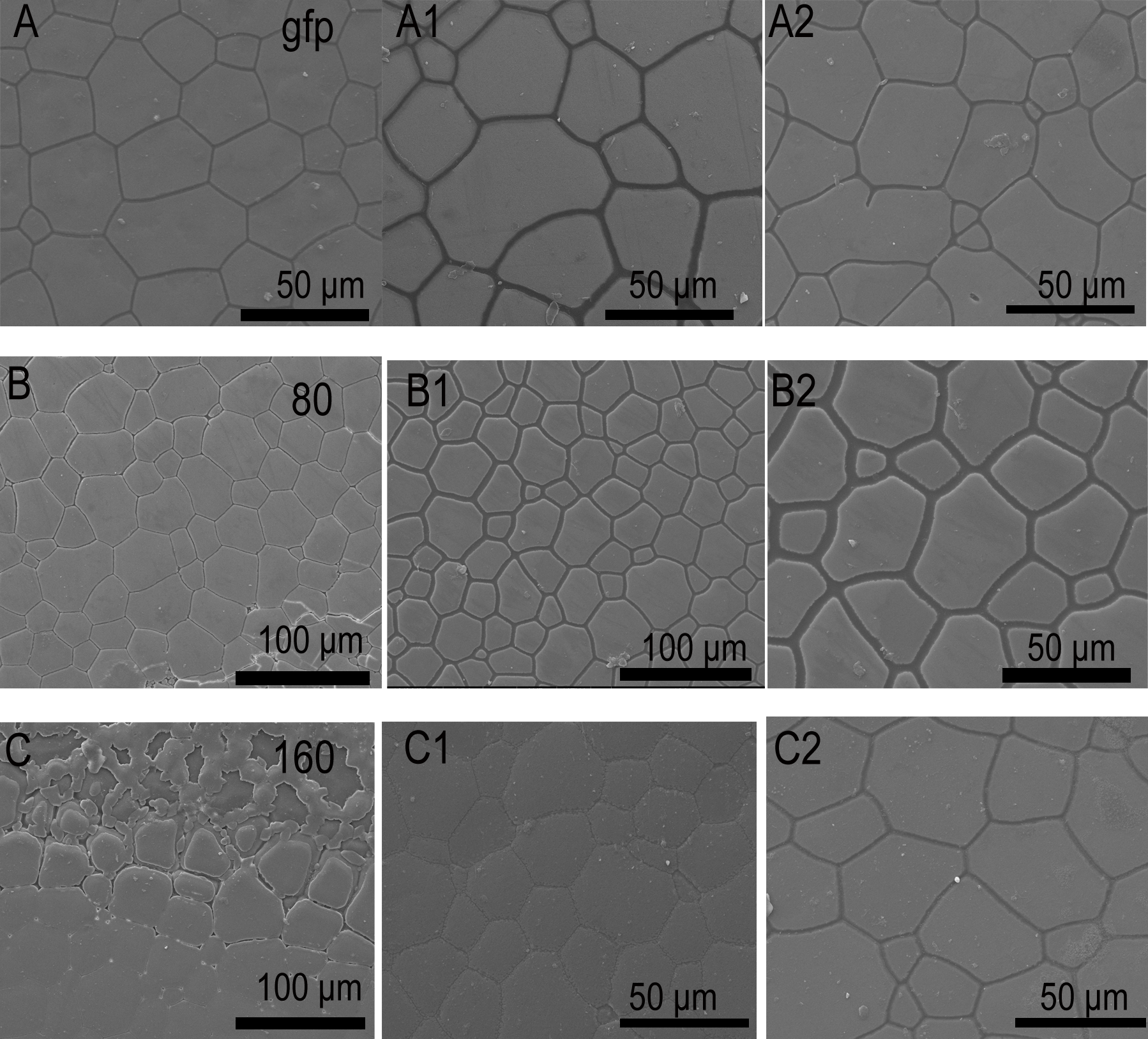


**Figure S3.** SEM images of prismatic layers. A-A2. the images of prismatic layers injected with *gfp* dsRNA-injected group. B-B2. the images of prismatic layers injected with 80 μg *fam20*C dsRNA-injected group. C-C2. The prismatic layers of 160 μg *fam20*C dsRNA-injected group.


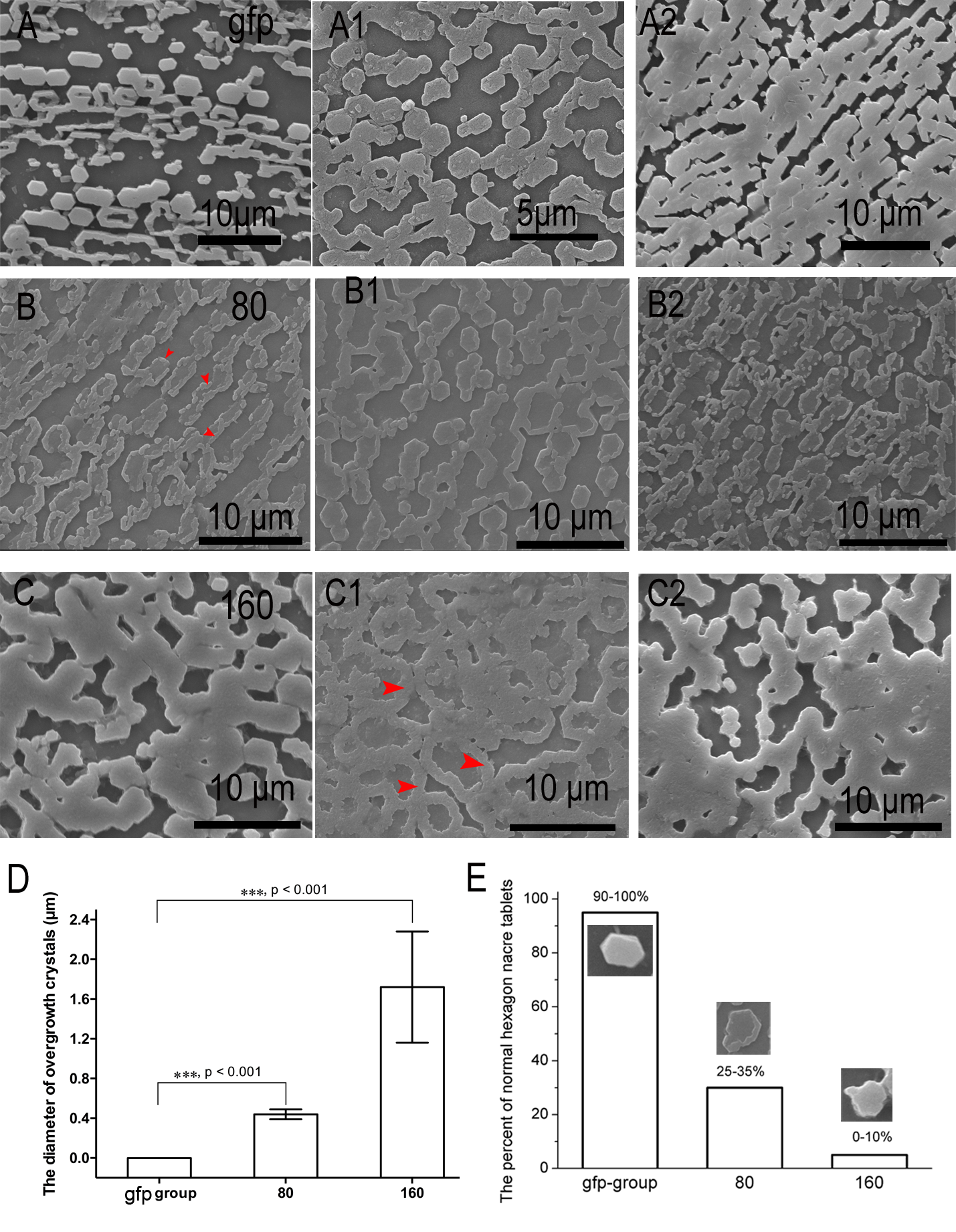


**Figure S4.** SEM images of nacreous layers. A-A2, the images of nacreous layers injected with *gfp* dsRNA-injected group. B-B2. the images of nacreous layers injected with 80 μg *fam20*C dsRNA-injected group. C-C2. The nacreous layers of 160 μg *fam20*C dsRNA-injected group. D. Comparison of the diameter of overgrowth crystals on nacre tablets (red arrows in B and C1).The stars represent a significant (***, p <0.001) difference compared with the *gfp* dsRNA-injected group. The error bars stand for stand deviation of statistics from three images. The diameter of overgrowth crystals on nacre tablets in 160 μg dsRNA-injected group were measured based on the diameter of nacre tablets in picture C and C2. E. The percent of normal hexagon nacre tablets in the *gfp* dsRNA-injected and *fam20*C dsRNA-injected groups. Normal hexagon nacre tablets refer to single tablets with clear boarders.

**Table S1. Primer sequences used in this study**

| Name | Sequence |
| --- | --- |
| Fam20C-3′RACE | CCACCCAGACACCGTAGAGGC |
| Fam20C-5′RACE | CGAGAAGGCAAGGCAAAGTAGTG |
| Fam20C-confirm-F | CGCTTTTTCAGGAATTATTACATGTT |
| Fam20C-confirm-R | TAAGCGGCTACCTTTTGTGGG |
| qFam20C-F | AACTACACCAGCGACGATTTCAGCC |
| qFam20C-R | CCCCCTCCTCCGATTTCTCTTCTTT |
| qGAPDH-F | GCCGAGTATGTGGTAGAATC |
| qGAPDH-R | CACTGTTTTCTGGGTAGCTG |
| Fam20C-F | AACTACACCAGCGACGATTTCAGCC |
| Fam20C-R | CCCCCTCCTCCGATTTCTCTTCTTT |
| dsFam20C-F | GCGTAATACGACTCACTATAGGGAGACTGGTCTAGCGAACTGGGAG |
| dsFam20C-R | GCGTAATACGACTCACTATAGGGAGAATTTTACGCTTGCCCATTTTC |
| dsGFP-F | GCGTAATACGACTCACTATAGGGAGAATGGTGAGCAAGGGCGAGGAG |
| dsGFP-R | GCGTAATACGACTCACTATAGGGAGATTACTTGTACAGCTCGTCCATG |
